# Investigation of Acid-Base Indicator Property of Plumbagin from *Plumbago zeylanica* Linn

**DOI:** 10.1155/2019/4061927

**Published:** 2019-08-18

**Authors:** Emmanuel B. A. Adusei, Reimmel K. Adosraku, James Oppong-Kyekyeku, Cedric D. K. Amengor

**Affiliations:** ^1^Department of Pharmaceutical Chemistry, Faculty of Pharmacy and Pharmaceutical Sciences, College of Health Sciences, Kwame Nkrumah University of Science and Technology, Kumasi, Ghana; ^2^Department of Pharmaceutical Chemistry, School of Pharmacy, University of Health and Allied Sciences, Ho, Ghana

## Abstract

There has been an increasing interest in the search for colour indicators of natural origin for titrimetric analysis. This is due to some challenges associated with the currently used synthetic ones. This study evaluates and validates the acid-base indicator property of plumbagin isolated from *Plumbago zeylanica* Linn. Plumbagin (5-hydroxy-2-methyl-1,4-naphthoquinone) was isolated from the roots of *Plumbago zeylanica* Linn using silica gel chromatography and characterized using spectroscopic methods in comparison with those reported in the literature. Its acid-base indicator property was evaluated alongside phenolphthalein and methyl orange, after it was found to exhibit a sharp change in colour at various pH ranges. The plumbagin indicator was successfully used to assay ibuprofen powder and tablets (400 mg) using the British Pharmacopoeia (2013) method. Data obtained were analyzed statistically by Student's *t*-test and one-way ANOVA in GraphPad Prism (version 5.01, 2010). Analysis of the use of the plumbagin indicator in acid-base titrations between strong acids and strong bases and between weak acids and strong bases has been evaluated and validated according to the ICH guidelines. Plumbagin use in ibuprofen powder and tablets has also been verified. Plumbagin has been validated for use as an indicator suitable for different acid-base titrations and the analysis of ibuprofen.

## 1. Introduction

Titrimetric analysis remains an important analytical technique in pharmaceutical analysis. It enables keen monitoring of the acid and base contents of raw materials, reaction mixtures, and resulting finished products in the industrial setting [1]. This method has several advantages including time and labour-saving, high precision, and determination of the purity of compounds in the absence of reference standards [1, 2]. In the past, this method has also been used to analyse and estimate the degraded products of drugs [2].

In acid-base titrations, acids are titrated with bases, and vice versa. They are also referred to as neutralization reactions, with the equivalent point detected either by use of colour indicators or potentiometrically with a glass electrode [3, 4]. They employ colour indicators to detect endpoints of acid-base neutralization reactions. Colour indicators show a sharp change in colour, in response to pH change in an acid-base titration reaction. Most indicators are either weak organic acids or basic dyes that accept or donate electrons [1, 5].

Acid-base indicators of natural origin have proven to be better alternatives in titrimetric analysis, as they are readily available, easy to extract, cheaper, less toxic, and environmentally friendly, unlike synthetic colour indicators which are associated with high cost and toxicity to the environment and users [4, 6, 7]. Plumbagin (5-hydroxy-2-methyl-1,4-naphthoquinone) is a natural organic dye found in plants of the families Plumbaginaceae, Droseraceae, Ebenaceae, Iridaceae, Ancistrocladaceae, Drosophyllaceae, and Nepenthaceae [8, 9].

Al-Nuri et al. [10] suggested that plumbagin may have acid-base indicator property in a study to spectrophotometrically quantify the compound in different parts of *Plumbago europaea* L. [10]. Hence, plumbagin isolated from *Plumbago zeylanica* Linn roots was evaluated for acid-base indicator property in this study. We hereby report our findings which reveal that plumbagin shows a prominent colour indicator property in acid-base titrations.

## 2. Materials and Methods

### 2.1. Materials and Chemicals

The materials used in the study included silica gel (70 : 230 mesh size) (Merck, US), silica gel-coated TLC plates (Merck, Germany), and ibuprofen BP powder and tablets (400 mg) (Ernest Chemists, Ghana). The chemicals employed were ethyl acetate (BDH Chemicals, UK), petroleum ether (VWR Chemicals, US), concentrated hydrochloric acid (36% w/v) (Surechem Products, UK), sodium hydroxide pellets (98% w/w) (Fisher Scientific, UK), Analar anhydrous sodium carbonate (99.5% w/w) (Surechem Products, UK), methanol (VWR Chemicals, US), Analar sulfamic acid (99% w/w) (Fisher Scientific, US), and acetic acid (Needham Market, Suffolk, UK).

### 2.2. Equipment

The equipment in this study included a melting point apparatus (UK/R000105350; Stuart), an analytical balance (electronic) (WD140050809; Kern, Germany), a Bruker FTIR spectrometer (Billerica, US), a UV spectrophotometer (7315; Jenway, UK), a Bruker Biospin NMR spectrometer (F/NMR/A 175; Billerica, US), a rotary evaporator (R-114; Buchi, Switzerland), a UV fluorescent lamp (UVP Inc, US), a pH meter (pH 211 microprocessor; Hanna Instruments), and a gravity column (90 cm × 6 cm with a fitted disc) (Merck, Germany).

### 2.3. Collection and Authentication of Plant Materials

Roots of *Plumbago zeylanica* Linn were collected in September 2018 from the Physic Garden of the Faculty of Pharmacy and Pharmaceutical Sciences, Kwame Nkrumah University of Science and Technology (KNUST). It was authenticated at the Department of Pharmacognosy, KNUST, with voucher number 003/10/07.

### 2.4. Isolation of Plumbagin

The powdered roots (2.4 kg) were cold macerated with ethyl acetate (10 L) for 5 days. The extract obtained on decantation was filtered by gravity using Whatman™ cellulose filter papers (1442-125, Grade 42, 2.5 *μ*, 12.5 cm), and the filtrate was concentrated with a rotary evaporator at a temperature of 40°C and controlled vacuum pressure, to a 2.5% w/w dry dark-brown solid mass.

The resulting slurry from the dry dark-brown solid mass for column chromatography was prepared by dissolving the dried extract in methanol, while adding small amount of silica. The mixture obtained was continuously stirred to allow for evaporation of the methanol to obtain a yellowish-orange solid product. The borosilicate chromatography column (90 cm × 6 cm) (Merck, Germany) with a fitted disc support was dried packed with silica (70 : 230 mesh size; stationary-phase material). Crude plumbagin (2.7 g) was eluted with a mobile-phase composition of ethyl acetate (15% v/v) in petroleum ether (85% v/v) followed by recrystallization from hot petroleum ether to obtain orange crystals (1.5 g). For the recrystallisation, crude plumbagin (2.7 g) was transferred into a conical flask (50 mL) followed by addition of petroleum ether. The mixture was heated to 60°C which dissolved the crude product completely. The resulting solution was allowed to stand at room temperature (28°C) which afforded gradual formation of needle-like crystals of plumbagin. The crystals were filtered by gravity using Whatman™ cellulose filter papers (1442-125, Grade 42, 2.5 *μ*, 12.5 cm) and allowed to dry at room temperature (28°C). The pure isolate's identity was established with melting point determination, 1D NMR (proton and carbon-13), and 2D NMR (COSY, HMBC, HSQC, DEPT-135) spectroscopy with the support of infrared (IR) and ultraviolet-visible (UV-Vis) spectroscopy techniques.

### 2.5. Screening of Plumbagin for Indicator Property

Plumbagin (0.05 g) was rendered completely soluble in methanol (50 mL) and diluted to the 100 mL mark with the same solvent in a volumetric flask to obtain a concentration of 0.05% w/v. Solutions of different pH (1–13) were prepared serially using 0.1 M·HCl, 0.1 M·NaOH, and distilled water (for pH 7) in test tubes. Plumbagin (5 drops) was added to the prepared solutions at room temperature, and observations were made for colour changes. The actual pH of the solutions where sharp colour changes were observed was determined with a calibrated pH meter as the working pH range of the plumbagin indicator.

### 2.6. Titrimetric Analysis (Colour Indicator)

Standard 0.1 M·HCl (10 mL) was titrated with standard 0.1 M·NaOH (strong acid/strong base) at room temperature using methyl orange as the standard indicator and the plumbagin solution under investigation. The endpoints from use of both indicators were determined visually. Replicate determinations were made, and the data obtained were statistically analyzed by one-way ANOVA in GraphPad Prism (Version 5.01, 2010).

Similarly, 0.1 M·CH_3_COOH solution (10 mL) was titrated with 0.1 M·NaOH (weak acid/strong base) using phenolphthalein as the standard indicator and the plumbagin solution under investigation. The endpoints were determined visually by colour changes of the indicators used. Replicate titrations were carried out, and the results were statistically analyzed by one-way ANOVA in GraphPad Prism (Version 5.01, 2010).

### 2.7. Assay of Ibuprofen BP Powder and Tablets

The British Pharmacopoeia method was used to assay ibuprofen BP powder (0.4500 g) and tablets (equivalent weight of powder containing 0.4500 g) [12]. The prepared ibuprofen solutions were titrated with standard 0.1 M·NaOH (titrant). Replicate analysis was carried out, and the obtained results were analyzed statistically by one-way ANOVA in GraphPad Prism (Version 5.01, 2010).

### 2.8. Validation of Indicator Property of Plumbagin

The use of the plumbagin indicator in titrations involving HCl/NaOH (strong acid/strong base) and CH_3_COOH/NaOH (weak acid/strong base) and in the assay of ibuprofen BP powder and tablets (400 mg) has been validated according to the ICH guidelines. The parameters evaluated were accuracy, precision (repeatability, interday precision, and intraday precision), robustness, and specificity.

## 3. Results and Discussion

### 3.1. Characterization of the Isolate (Plumbagin)

Pure plumbagin (1.5 g) was obtained as orange needle-like crystals with a melting point of 78–80°C. The retardation factor (*R*_f_) was obtained on a silica gel-coated TLC plate developed with the mobile phase of petroleum ether-ethyl acetate (70% v/v : 30% v/v) as 0.82.

The UV-visible spectrum showed two absorption maxima at 265 nm and 420 nm, identical to that reported by Annan et al. [11]. The IR spectrum revealed presence of a broad band at 3293 cm^−1^ indicative of an OH group. Strong peaks observed at 1161.60 cm^−1^ and 1639.66 cm^−1^ are indicative of free and hydrogen-bonded carbonyls, respectively. Vibrations at 1605.48 cm^−1^ are due to aromatic C=C. A weak absorption at 3038.4 cm^−1^ indicates –C-H stretches of sp^2^ hybridized carbons of an aromatic system. Vibration occurring at 1227.90 cm^−1^ is indicative of a phenolic CO group.

In the ^1^H NMR (CDCl_3_) spectrum, a doublet peak occurred at *δ* 2.18 (3H, d) and is due to the three protons of the methyl group present. A singlet peak at *δ* 6.79 is assigned to the quinoid ring proton at position C-3. Multiplet signals observed at *δ* 7.24–7.61 (3H, m) resulted from the three aromatic protons at positions C-6, C-7, and C-8, respectively. A singlet peak occurring at *δ* 11.94 (1H, s) is attributed to the intramolecularly hydrogen-bonded phenolic proton.

The ^13^C NMR (CDCl_3_) spectrum revealed a total of 11 carbons in the compound. Two peaks occurring at *δ* 184.73 and *δ* 190.24 are indicative of a free carbonyl (C-1) and hydrogen-bonded carbonyl (C-4), respectively.

**Figure 1:**
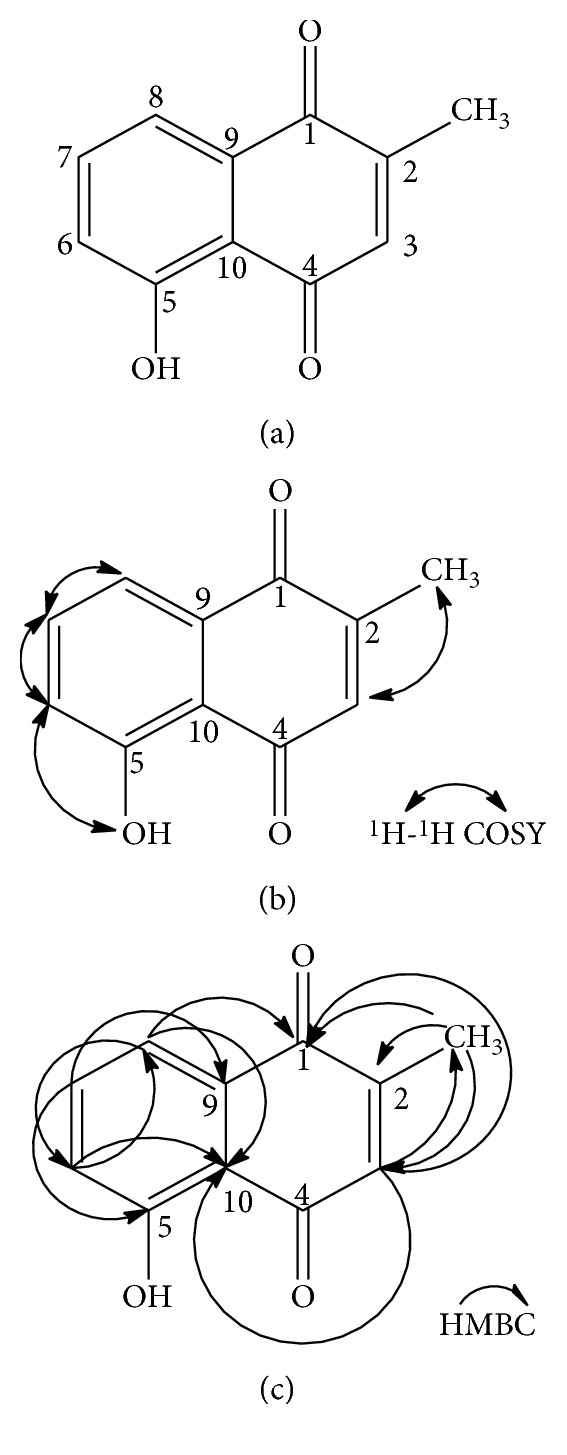
(a) Plumbagin. (b) ^1^H-^1^H COSY of plumbagin. (c) Key HMBC correlations of plumbagin.

A peak occurring at *δ* 161.22 indicated a phenolic quaternary carbon (C-6). Peaks occurring at *δ* 115.17, *δ* 132.13, and *δ* 149.61 resulted from quaternary olefinic carbons at positions C-10, C-9, and C-2, respectively. The spectrum also shows four aromatic methine peaks occurring at *δ* 135.45 (C-3), *δ* 124.13 (C-6), *δ* 136.05 (C-7), and *δ* 119.24 (C-8), respectively. A methyl peak occurred at *δ* 16.40 (C-11). The peak obtained at *δ* 161.22 is attributed to the oxygen-bearing quaternary carbon (C-5) (Figure 1). Various correlations existing in the compound were confirmed with two-dimensional (2D) NMR techniques (COSY and HMBC), as shown in Figure 1.

### 3.2. Acid-Base Titrimetry

**Figure 2:**
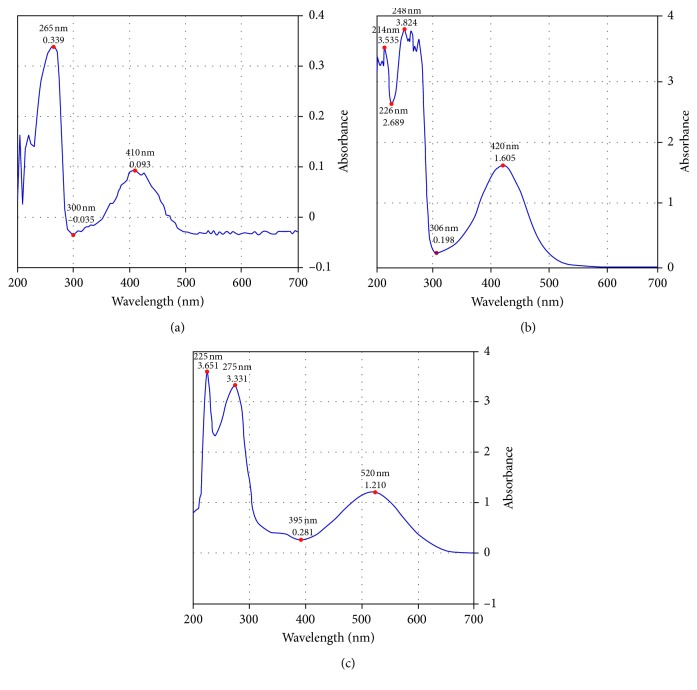
UV spectra for plumbagin in different pH media. (a) Plumbagin in methanol. Absorption occurs at 265 nm (*λ*_max_) and 410 nm. (b) Plumbagin in acidified methanol. Absorption takes place at 248 nm (*λ*_max_) and 420 nm from hypsochromic shift. (c) Plumbagin in basified methanol. Absorption takes place at 275 nm (*λ*_max_) and 520 nm from bathochromic shift.

Plumbagin indicator solution of 0.05% w/v concentration was prepared in methanol (Figure 2(a)). Colour changes observed in different pH solutions resulted in identification of plumbagin as an indicator. Drops of methanolic solution of plumbagin gave a yellow colouration in the acidic medium and pink colour in the basic or alkaline medium. The phenolic OH group attached to the naphthoquinone nucleus is protonated in the acidic medium, leading to the reduction of electron cloud density, which results in absorption at wavelengths of 248 nm (*λ*_max_) and 420 nm (hypsochromic shift and hypochromic effect) (Figure 2(b)). Alternatively, the phenolic OH is ionized in the alkaline medium (1), resulting in the increase in electron cloud density and absorption at 275 nm (*λ*_max_) and 520 nm (bathochromic shift and hyperchromic effect) (Figure 2(c)). The working pH range of the plumbagin indicator was determined to be 8.02–10.07.

Statistical analysis using one-way ANOVA showed no significant difference in the results obtained from use of methyl orange and plumbagin in titration of HCl with NaOH (*p*=0.7730). Similarly, no significant difference is seen with the results obtained from use of phenolphthalein and plumbagin in titrations of CH_3_COOH with NaOH (*p*=0.0896) (Table 1).

Indicator errors of the two standard indicators as well as plumbagin were determined under similar conditions. Statistical analysis by Student's *t*-test did not show a significant difference between methyl orange and plumbagin. No significant difference was observed with indicator errors of phenolphthalein and plumbagin as well.

### 3.3. Validation of Use of Plumbagin Indicator

**Figure 3:**
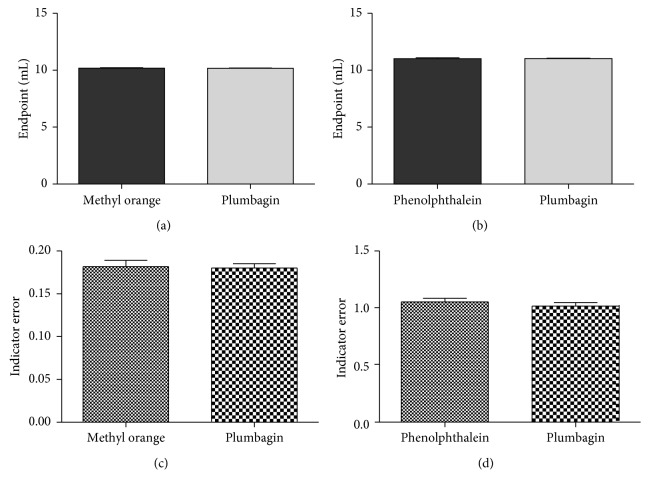
Mean ± SEM of reaction endpoints and indicator errors from titrations with plumbagin, methyl orange, and phenolphthalein. (a) Mean ± SEM of endpoint for titration of HCl with NaOH. (b) Mean ± SEM of endpoint for titration of CH_3_COOH with NaOH. (c) Indicator errors of plumbagin and methyl orange in titration of HCl with NaOH. (d) Indicator errors of plumbagin and phenolphthalein in titration of CH_3_COOH with NaOH. Each bar represents mean ± SEM of endpoints/indicator errors; SEM: standard error of mean.

The accuracy of an analytical method is the closeness of the test results obtained to the mean or the theoretical true value [1]. The accuracy of the plumbagin indicator was determined in titrations involving 0.1 M·HCl/0.1 M·NaOH and 0.1 M·CH_3_COOH/0.1 M·NaOH, by comparing endpoint results obtained with those from use of methyl orange and phenolphthalein, respectively. Statistical analysis of endpoints using a two-tailed Student's *t*-test (in GraphPad Prism) showed no significant difference in endpoint values obtained with the use of plumbagin and methyl orange indicators (*p*=0.7730) (Figure 3(a)). No significant difference was also observed with the use of plumbagin and phenolphthalein indicators (*p*=0.0896) (Figure 3(b)). The indicator errors of plumbagin and the standard indicators were also compared statistically using Student's *t*-test at a confidence interval of 95% in further evaluation of accuracy. The results showed no significant difference in indicator error compared to those of methyl orange (*p*=0.9674) (Figure 3(c)) and phenolphthalein (*p*=0.0896) (Figure 3(d)). Accuracy was further evaluated with percentage recovery obtained from the assay of ibuprofen powder using plumbagin as an indicator. The percentage recovery ranged between 98.80% and 100.81%, with a mean of 99.94%. The obtained results fall within the acceptance criteria of 98.0%–102.0% (Table 2).

Precision refers to the closeness of agreement of results under defined conditions. It is expressed as standard deviation (SD) or relative standard deviation (RSD) [13]. Evaluation of precision by repeatability was achieved using plumbagin (0.10%) in titrations between 0.1 M·HCl and 0.1 M·NaOH and between 0.1 M·CH_3_COOH and 0.1 M·NaOH. The RSD for ten replicate determinations was obtained to be 0.48% and 0.61%, respectively, which agrees with the acceptance criteria ≤2.0% (Table 3). The RSD obtained for percentage purity and content of ibuprofen powder and tablets (400 mg) was 0.41% and 0.68%, respectively, which also agrees with the acceptance criterion.

Evaluation of precision by intermediate precision was achieved through statistical analysis of results obtained by the same analyst on different experimental days (interday precision) and results obtained by the same analyst on different experimental times (intraday precision). The RSD obtained for both interday and intraday precision results was found to comply with the acceptance criteria ≤2.0%. Statistical analysis by one-way ANOVA showed no significant differences (*p* ≥ 0.05) (Tables 4 and 5).

The robustness of an analytical method refers to the degree of reproducibility of test results obtained from same samples under varied or altered conditions [3]. This was evaluated using 50%, 100%, and 150% plumbagin, representing 0.05%, 0.10%, and 0.15%, respectively, with different drops of a particular concentration of plumbagin, and by different analysts on the same experimental day. The RSD of the results obtained in all the analysis complied with acceptance criteria ≤2.0%. Statistical analysis of results obtained by one-way ANOVA failed to produce significantly different results on alteration of test conditions (*p* ≥ 0.05) (Tables 6–8).

Specificity is the ability to unequivocally assess an analyte in the presence of expected components [14]. This was achieved through the establishment of presence of plumbagin in the indicator solution and attribution of titration endpoints to plumbagin, and not the solvent used to prepare the indicator solution (methanol). Five drops of plumbagin and methyl orange indicators were added to volumes (10 mL ± 0.1) of 0.1 M·NaOH, 0.1 M·HCl, and 0.1 M·CH_3_COOH in separate conical flasks. The solutions were titrated with 0.1 M·HCl and 0.1 M·NaOH, respectively, and colour changes were observed. Methanol did not affect colour changes in the analyte solutions, and as such, endpoints of the reactions between the titrants and analytes could not be monitored visually (Table 9). Colour changes therefore observed in the titrimetric analysis were attributed solely to the plumbagin indicator.

**Figure 4:**
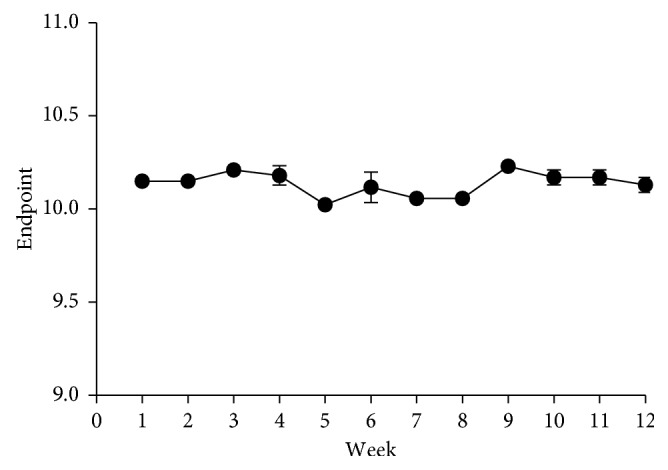
Endpoints obtained from a three-month study of use of freshly prepared 0.05% plumbagin solution.

Stability of a colour indicator solution enables the prediction of the period within which the indicator is fit for its intended purpose or use [1]. The stability of plumbagin was determined with freshly prepared 0.05% indicator solution in titration of 0.1 M·HCl/0.1 M·NaOH over a 3-month duration with replicate determinations on weekly basis. The RSDs for all the determinations complied with the acceptance criteria ≤2.0% (Figure 4).

## 4. Conclusion

The acid-base indicator property of plumbagin has been evaluated and validated in accordance with the ICH guidelines. Plumbagin which was easily extracted from its source was found to play a dual role as an alternative indicator to methyl orange and phenolphthalein in titrations involving strong acids/strong bases and weak acids/strong bases. Hence, it can be used as an indicator in analytical laboratories and could be a suitable substitute for methyl orange and phenolphthalein for such titrations hitherto mentioned in this study.

## Figures and Tables

**Scheme 1:**
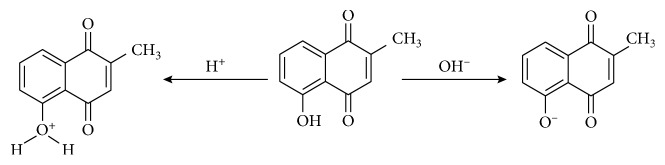
Effects of pH changes on the plumbagin indicator.

**Table 1:** Comparison of results obtained using standard indicators and plumbagin.

HCl/NaOH (mL)		CH_3_COOH/NaOH (mL)		Assay of ibuprofen powder (% purity)		Assay of ibuprofen BP tablets (% content)	
Methyl orange	Plumbagin	Phenolphthalein	Plumbagin	Phenolphthalein	Plumbagin	Phenolphthalein	Plumbagin
10.27	10.18	11.10	11.00	99.50	98.80	97.45	97.30
10.18	10.18	11.10	11.08	100 00	99.30	95.30	96.78
10.18	10.18	11.10	11.08	100 00	98.80	95.00	95.53
10.18	10.27	11.08	11.08	99.50	99.80	97.50	97.10
10.27	10.09	11.08	11.00	99 00	99.30	99.02	96.82
9.99	10.09	10.90	10.90	99.5 0	99.80	96.30	97.25
Mean = 10.18 ± 0.102	Mean = 10.17 ± 0.068	Mean = 11.06 ± 0.079	Mean = 11.02 ± 0.072	Mean = 99.58 ± 0.376	Mean = 99.30 ± 0.447	Mean = 96.76 ± 1.521	Mean = 96.80 ± 0.657
RSD = 1.00%	RSD = 0.67%	RSD = 0.71%	RSD = 0.65%	RSD = 0.38%	RSD = 0.45%	RSD = 1.57%	RSD = 0.68%
*p*=0.7730		*p*=0.0896		*p*=0.3442		*p*=0.9498	

**Table 2:** Percentage recovery of ibuprofen BP using plumbagin as an indicator.

Percentage purity		
Phenolphthalein	Plumbagin	Percentage recovery
99.50	98.80	99.30
100.00	99.30	99.30
100.00	98.80	98.80
99.50	99.80	100.30
99.00	99.30	100.30
99.50	99.80	100.30
99.00	99.80	100.81
100.00	99.80	99.80
99.50	99.80	100.30
100.00	99.80	99.80
99.00	99.30	100.30
Mean ± SEM = 99.94		
Acceptance criteria = 98%–102%		

SEM: standard error of mean.

**Table 3:** Evaluation of repeatability.

HCl/NaOH (mL)	CH_3_COOH/NaOH (mL)	Assay of ibuprofen powder (% purity)	Assay of ibuprofen BP tablets (% content)
10.18	11.00	98.80	97.30
10.18	11.08	99.30	96.78
10.18	11.08	98.80	95.53
10.27	11.08	99.80	97.10
10.09	11.00	99.30	96.82
10.09	10.90	99.80	97.25
10.18	10.90	99.80	97.82
10.18	11.00	99.80	97.87
10.18	11.00	99.80	96.80
10.18	11.00	99.80	97.67
10.18	11.08	99.30	96.90
Mean = 10.17 ± 0.049	Mean = 11.01 ± 0.067	Mean = 99.48 ± 0.405	Mean = 96.64 ± 0.653
RSD = 0.48%	RSD = 0.61%	RSD = 0.41%	RSD = 0.68%

**Table 4:** Effects of the indicator on the interday precision of the method.

HCl/NaOH (mL)			CH_3_COOH/NaOH (mL)			Assay of ibuprofen powder (% purity)			Assay of ibuprofen BP tablets (% content)		
Day 1	Day 2	Day 3	Day 1	Day 2	Day 3	Day 1	Day 2	Day 3	Day 1	Day 2	Day 3
10.18	10.21	10.11	11.00	11.04	11.04	98.80	100.12	100	96.14	96.42	98.00
10.18	10.21	10.11	11.08	11.20	11.04	99.30	99.64	99.58	95.98	97.24	98.58
10.18	10.21	10.11	11.08	11.20	11.04	98.80	99.64	99.58	98.50	97.57	97.61
10.27	10.11	10.11	11.08	11.20	11.04	99.80	99.64	99.58	98.30	97.26	98.35
10.09	10.11	10.21	11.00	11.26	11.04	99.30	100.12	100	96.00	96.77	97.30
10.09	10.11	10.21	10.90	11.20	10.93	99.80	99.64	100	97.10	97.10	97.11
Mean = 10.17 ± 0.068	Mean = 10.16 ± 0.055	Mean = 10.14 ± 0.052	Mean = 11.02 ± 0.072	Mean = 11.02 ± 0.045	Mean = 11.02 ± 0.045	Mean = 99.30 ± 0.447	Mean = 99.80 ± 0.248	Mean = 99.79 ± 0.230	Mean = 97.00 ± 1.160	Mean = 97.06 ± 0.407	Mean = 97.83 ± 0.585
RSD = 0.67%	RSD = 0.54%	RSD = 0.51%	RSD = 0.65%	RSD = 0.66%	RSD = 0.41%	RSD = 0.45%	RSD = 0.25%	RSD = 0.23%	RSD = 1.20%	RSD = 0.42%	RSD = 0.60%
*p*=0.8005			*p*=0.5284			*p*=0.0764			*p*=0.1639		

**Table 5:** Effects of the indicator on the intraday precision of the method.

HCl/NaOH (mL)			CH_3_COOH/NaOH (mL)			Assay of ibuprofen powder (% purity)			Assay of ibuprofen BP tablets (% content)		
Morning	Afternoon	Evening	Morning	Afternoon	Evening	Morning	Afternoon	Evening	Morning	Afternoon	Evening
10.18	10.18	10.18	11.00	11.14	11.09	98.80	99.64	100.63	96.00	97.70	99.60
10.18	10.18	10.09	11.08	11.25	11.09	99.30	99.64	100.63	97.82	98.50	98.51
10.18	10.18	10.09	11.08	11.25	10.98	98.80	100.10	100.10	96.87	98.30	99.22
10.27	10.09	10.09	11.08	11.25	10.98	99.80	100.10	100.63	96.80	97.80	98.40
10.09	10.09	10.18	11.00	11.14	10.98	99.30	99.64	100.10	97.67	97.00	98.92
10.09	10.09	10.09	10.90	11.25	10.90	99.80	99.64	100.10	96.90	99.30	98.43
Mean = 10.17 ± 0.028	Mean = 10.14 ± 0.049	Mean = 10.12 ± 0.019	Mean = 11.02 ± 0.029	Mean = 11.21 ± 0.023	Mean = 11.00 ± 0.030	Mean = 99.30 ± 0.183	Mean = 99.79 ± 0.097	Mean = 100.40 ± 0.119	Mean = 97.01 ± 0.270	Mean = 98.10 ± 0.322	Mean = 98.85 ± 0.200
RSD = 0.67%	RSD = 0.49%	RSD = 0.46%	RSD = 0.65%	RSD = 0.51%	RSD = 0.67%	RSD = 0.45%	RSD = 0.24%	RSD = 0.29%	RSD = 0.68%	RSD = 0.80%	RSD = 0.50%
*p*=0.3811			*p*=0.1100			*p*=0.1230			*p*=0.1800		

**Table 6:** Effects of the indicator concentration on the method robustness.

HCl/NaOH (mL)			CH_3_COOH/NaOH (mL)			Assay of pure ibuprofen powder (% purity)			Assay of ibuprofen BP tablets (% content)		
0.05%	0.10%	0.15%	0.05%	0.10%	0.15%	0.05%	0.10%	0.15%	0.05%	0.10%	0.15%
9.99	10.09	10.18	10.90	11.08	11.00	100.30	99.30	98.80	99.98	96.00	97.30
9.99	10.18	10.18	11.00	11.10	11.08	100.30	99.80	99.30	97.10	97.82	96.78
9.99	9.99	10.18	10.90	11.00	11.08	99.80	99.80	98.80	98.67	96.87	95.53
10.09	9.99	10.27	10.90	10.90	11.08	100.30	100.30	99.80	97.50	96.80	97.10
9.99	10.09	10.09	11.00	11.00	11.00	99.80	100.30	99.30	99.50	97.67	96.82
10.09	9.99	10.09	11.08	11.31	10.90	99.80	100.30	99.80	98.50	96.90	97.25
Mean = 10.02 ± 0.021	Mean = 10.06 ± 0.032	Mean = 10.17 ± 0.028	Mean = 10.96 ± 0.030	Mean = 11.07 ± 0.057	Mean = 11.02 ± 0.029	Mean = 100.10 ± 0.112	Mean = 99.97 ± 0.167	Mean = 99.30 ± 0.183	Mean = 98.54 ± 0.454	Mean = 97.01 ± 0.270	Mean = 96.80 ± 0.268
RSD = 0.52%	RSD = 0.78%	RSD = 0.67%	RSD = 0.69%	RSD = 1.26%	RSD = 0.65%	RSD = 0.27%	RSD = 0.41%	RSD = 0.45%	RSD = 1.13%	RSD = 0.68%	RSD = 0.68%
*p*=0.7818			*p*=0.2439			*p*=0.0800			*p*=0.1000		

**Table 7:** Effects of the indicator drops on the method robustness.

HCl/NaOH (mL)			CH_3_COOH/NaOH (mL)			Assay of ibuprofen powder (% purity)			Assay of ibuprofen BP tablets (% content)		
3 drops	5 drops	7 drops	3 drops	5 drops	7 drops	3 drops	5 drops	7 drops	3 drops	5 drops	7 drops
10.18	9.99	10.09	11.00	11.08	11.08	98.80	100.30	99.80	99.20	96.00	96.14
10.18	10.09	10.18	11.08	10.90	11.08	99.30	99.80	99.80	98.44	97.82	95.98
10.18	10.09	10.18	11.08	11.00	11.08	98.80	100.30	99.80	98.00	96.87	98.50
10.27	10.09	10.09	11.08	10.90	11.08	99.80	100.30	99.80	96.29	96.80	98.30
10.09	10.18	9.99	11.00	11.00	11.00	99.30	99.80	99.30	96.16	97.67	96.00
10.09	10.18	10.18	10.90	11.08	11.00	99.80	99.80	100.30	97.00	96.90	97.10
Mean = 10.17 ± 0.028	Mean = 10.10 ± 0.029	Mean = 10.12 ± 0.031	Mean = 11.02 ± 0.029	Mean = 10.99 ± 0.033	Mean = 11.05 ± 0.017	Mean = 99.30 ± 0.183	Mean = 100.1 ± 0.112	Mean = 99.80 ± 0.129	Mean = 97.52 ± 0.501	Mean = 97.01 ± 0.270	Mean = 97.00 ± 0.474
RSD = 0.67%	RSD = 0.29%	RSD = 0.31%	RSD = 0.26%	RSD = 0.30%	RSD = 0.15%	RSD = 0.18%	RSD = 0.11%	RSD = 0.13%	RSD = 0.51%	RSD = 0.28%	RSD = 0.49%
*p*=0.3285			*p*=0.3260			*p*=0.071			*p*=0.6331		

**Table 8:** Different analysts on the same experimental day for evaluation of robustness.

HCl/NaOH (mL)		CH_3_COOH/NaOH (mL)		Assay of pure ibuprofen powder (% purity)		Assay of ibuprofen BP tablets (% content)	
Analyst 1	Analyst 2	Analyst 1	Analyst 2	Analyst 1	Analyst 2	Analyst 1	Analyst 2
10.18	10.09	11.00	10.82	98.80	99.90	96.14	99.11
10.18	9.99	11.08	11.05	99.30	100.40	95.98	98.80
10.18	10.09	11.08	10.93	98.80	100.40	98.50	98.70
10.27	10.09	11.08	11.05	99.80	99.90	98.30	97.98
10.09	10.18	11.00	11.05	99.30	100.40	96.00	98.50
10.09	9.99	10.90	11.05	99.80	100.90	97.10	99.30
Mean = 10.17 ± 0.028	Mean = 10.07 ± 0.029	Mean = 11.02 ± 0.029	Mean = 10.99 ± 0.040	Mean = 99.30 ± 0.183	Mean = 100.3 ± 0.376	Mean = 97.00 ± 0.474	Mean = 98.73 ± 0.191
RSD = 0.67%	RSD = 0.72%	RSD = 0.65%	RSD = 0.88%	RSD = 0.45%	RSD = 0.38%	RSD = 1.20%	RSD = 0.47%
*p*=0.0721		*p*=0.5561		*p*=0.0900		*p*=0.640	

**Table 9:** Colour of the indicator and solvent in different solutions on evaluation of specificity.

	Plumbagin			Methanol		
	NaOH (analyte)/HCl (titrant)	HCl (analyte)/NaOH (titrant)	CH_3_COOH (analyte)/NaOH (titrant)	NaOH (analyte)/HCl (titrant)	HCl (analyte)/NaOH (titrant)	CH_3_COOH (analyte)/NaOH (titrant)
Colour of the indicator before titration	Pink	Yellow	Yellow	Colourless	Colourless	Colourless
Colour of the indicator after titration	Yellow	Pink	Pink	Colourless	Colourless	Colourless

## Data Availability

The data are available from the Pharmaceutical Chemistry Laboratory, Faculty of Pharmacy, KNUST, Ghana. However, the data used to support the findings of this research are within this article.
